# Tissue and serum expression of TGM-3 may be prognostic marker in patients of oral squamous cell carcinoma undergoing chemo-radiotherapy

**DOI:** 10.1371/journal.pone.0199665

**Published:** 2018-06-28

**Authors:** Seema Nayak, M. L. B. Bhatt, Madhu Mati Goel, Seema Gupta, Abbas Ali Mahdi, Anupam Mishra, Divya Mehrotra

**Affiliations:** 1 Department of Radiotherapy, King George’s Medical University Lucknow, Uttar Pradesh, India; 2 Department of Pathology, King George’s Medical University Lucknow, Uttar Pradesh, India; 3 Department of Biochemistry, King George’s Medical University Lucknow, Uttar Pradesh, India; 4 Department of Otorhinolaryngology, King George’s Medical University Lucknow, Uttar Pradesh, India; 5 Department of Oral and Maxillofacial Surgery, King George’s Medical University Lucknow, Uttar Pradesh, India; University of South Alabama Mitchell Cancer Institute, UNITED STATES

## Abstract

Radioresistance is one of the main determinants of treatment outcome in oral squamous cell carcinoma (OSCC), but its prediction is difficult. Several authors aimed to establish radioresistant OSCC cell lines to identify genes with altered expression in response to radioresistance. The development of OSCC is a multistep carcinogenic process that includes activation of several oncogenes and inactivation of tumour suppressor genes. TGM-3 is a tumour suppressor gene and contributes to carcinogenesis process. The aim of this study was to estimate serum and tissue expression of TGM-3 and its correlation with clinico-pathological factors and overall survival in patients of OSCC undergoing chemo-radiotherapy. Tissue expression was observed in formalin fixed tissue biopsies of 96 cases of OSCC and 32 healthy controls were subjected to immunohistochemistry (IHC) by using antibody against TGM-3 and serum level was estimated by ELISA method. mRNA expression was determined by using Real-Time PCR. Patients were followed for 2 year for chemo radiotherapy response. In OSCC, 76.70% cases and in controls 90.62% were positive for TGM-3 IHC expression. TGM-3 expression was cytoplasmic and nuclear staining expressed in keratinized layer, stratum granulosum and stratum spinosum in controls and tumour cells. Mean serum TGM-3 in pre chemo-radiotherapy OSCC cases were 1304.83±573.55, post chemo-radiotherapy samples were 1530.64±669.33 and controls were 1869.16±1377.36, but difference was significant in pre chemo-radiotherapy samples as compared to controls (p<0.018). This finding was also confirmed by real- time PCR analysis in which down regulation (-7.92 fold change) of TGM-3 in OSCC as compared to controls. TGM-3 expression was significantly associated with response to chemo-radiotherapy treatment (p<0.007) and overall survival (p<0.015). Patents having higher level of TGM-3 expression have good response to chemo-radiotherapy and also have better overall survival. TGM-3 may serve as a candidate biomarker for responsiveness to chemo-radiotherapy treatment in OSCC patients.

## Introduction

Oral squamous cell carcinoma (OSCC) is the sixth most prevalent cancer worldwide and is the leading causes for cancer-related deaths [1]. There are improvement in its detection, prevention and treatment in last decades; outcome and prognosis related to cure and survival have still been poorer due to treatment resistance and tumour recurrence. The 5- year survival of 50%, has remained disappointingly stable over the last few decades in spite of improvements in the main treatments of surgery and radiotherapy [2–4]. These treatments are often toxic and can affect long term functioning and quality of life of the patients [5]. So there is a need to identify biomarkers that are able to predict prognosis and response to treatment [5]. The most informative marker is node status, N-status, where cervical lymph node involvement drastically worsens the prognosis.

High failure rate and low median survival rate are observed in patients undergoing conventional chemo-radiotherapy with recurrent, intractable OSCC. More than 30% of patients eventually develop local recurrence or metastasis usually within the first 2 year of follow-up and completion of treatment [6]. Identification of biomarker can help tailor therapy on an individual basis and reduce treatment–related toxicity [5].

Radioresistance is the major contributor of radiotherapy treatment failure in OSCC, and its prediction is also very difficult. There are several studies that establish radioresistant OSCC cell lines to identify genes with altered expression in response to radioresistance [7].

The development of OSCC is a multistep carcinogenic process that includes activation of several oncogenes and inactivation of tumour suppressor genes [8]. Transglutaminases (TGMs) are a family of calcium dependent enzymes catalyze the formation of isopeptide bonds [9–10]. TGM-3 is epidermal transglutaminase and expressed predominantly in the suprabasal layers of the stratified squamous epithelium [11]. TGM-3 gene is widely expressed in the small intestine, brain, skin and mucosa [12]. In the skin and mucosa, TGM-3 expressed in the suprabasal layers of the stratified squamous epithelium [13–14]. TGM-3 is essential for epidermal terminal differentiation and formation of the cornified cell envelope through cross-linking structural proteins such as involucrin, loricrin and small proline-rich proteins [15–16]. Several studies showed that the down-regulation of the TGM-3 gene is associated with a variety of human cancer types, including laryngeal carcinoma, esophageal and OSCC [17–19].

Wu et al. [20] prove that TGM-3 as a candidate tumour suppressor gene and contributes to the carcinogenesis and development of Head and neck squamous cell carcinoma (HNSCC).

So the aim of our study was to estimate serum and tissue expression of TGM-3 and its correlation with clinico-pathological factors, response to chemo-radiotherapy treatment and overall survival in patients of OSCC. Hence this study was to evaluate either TGM-3 may be used as biomarkers for chemo-radiotherapy resistant or sensitive in OSCC patients.

## Materials and methods

### Subjects and sample collection

Tissue biopsies were obtained from cases of 96 OSCC and 32 healthy controls from the Departments of Otorhinolaryngology and Oral and Maxillofacial Surgery, King George’s Medical University Lucknow after obtaining the institutional ethical approval and informed written consent from patients during the years 2015 to 2017. Ethical approval obtained from King George's Medical University U.P. Institutional ethical committee registration No. EcR/262/Inst/UP/2013 approved this study with reference code: 72nd ECM II-B Fellowship/P12 dated 29th June 2015.Healthy oral tissues were obtained from patients undergoing cosmetic surgery, who did not have any infective or inflammatory oral lesion.

### Clinical assessment

Detailed clinical history including age, sex, symptoms, duration of illness, adverse oral habits like smoking or chewing of tobacco, alcohol consumption, clinical detail of the lesion, histological grading, clinical staging, course of treatment, outcome and recurrence was recorded on a detailed pre-tested structured proforma. Tumour (T) stage, nodal (N) status and TNM stage was classified according to the 1997 American Joint Committee on Cancer (AJCC) system.

Follow up was done to evaluate chemo-radiation response and overall survival. The patients were followed-up every 2 months in the 1st year, every 3 months in the 2nd year. The median follow up time was 10 months. Overall survival was measured from the date of histological diagnosis to death or last follow-up.

### Chemo- radiotherapy assessment

All the patients were given 2-cycles of induction taxol (175mg/m2 day 1) and cisplatin (50 mg/m2 day2) chemotherapy and they were subjected for radiation along with concurrent cisplatin (35mg/m2) 4-weeks from completion of induction chemotherapy. Radiotherapy was given by External beam Conventional Method (200CGy/fraction) to a total dose of 70 Gy in 35 fractions in 7- weeks by cobalt^60^ to primary tumors site and neck. The protocol plan was continued despite mucositis or dermatitis. However, the dose of cisplatin was reduced to 50% if the calculated creatinine clearance level was 30–50 ml/min. No cisplatin was given if the creatinine clearance level was less than 30ml/min. In presence of myelo supression (WBC count < 4000/mm3 or platelets count less than 100,000/mm3), persistent fever that exceeded 38°C or other clinically apparent infections, chemoradiation was postponed for1-week or interrupted. Synchronous chemotherapy in the form of injection cisplatinum 30 mg/ml weekly was delivered with adequate hydration, diuresis and anti-emetic prophylaxis.

Patients were evaluated for response to treatment one month after the completion of radiotherapy or chemo-radiotherapy. The response in primary tumors was evaluated using WHO criteria. Complete response (CR) was defined as the disappearance of the tumor; partial response (PR), a reduction of >50% of tumor and rest of the patients with neither CR nor PR were considered as non-responder (NR). CR and PR patients were considered as responders and patients with stable disease (SD) or progressive disease (PD) were classified as clinical non-responders (NR).

### Histopathological examination

All tissues were fixed in 10% neutral buffered formalin and processed for histopathological examination as per standard procedure. 5μm thick sections were cut and stained with haematoxylin and eosin (H&E). Sections were reviewed by two independent pathologists and histological diagnosis was made as per WHO criteria.

### Immunohistochemistry

Sections were deparaffinised in xylene followed by hydration in descending ethanol grades. Antigen retrieval was performed by heating specimens for 15 min at 95°C in citrate buffer (pH 6.0) using an EZ antigen retriever system (BioGenex, USA). Then neutralize endogenous peroxidise by using peroxidise block for 5 minutes. After washing with Tris buffer saline (TBS; pH 7.4) sections were incubated with protein block for 5 minutes. After TBS washing 2 times sections were incubated overnight at 4°C with primary Rabbit polyclonal antibodies against human TGM-3 (Biorbyt Ltd. United Kingdom). Primary antibody detected using polymer based Novolink secondary kit. After thorough washing with TBS sections were treated with post primary for 30 minutes at room temperature followed by incubation with Novolink polymer for 30 min at room temperature. After three washes with TBS, DAB substrate (3,3’-diaminobenzidine tetrahydrochloride) was applied to the sections for 5–10 min in the dark. Sections were counterstained with hematoxylin, dehydrated with ascending ethanol grades and xylene and mounted permanently with DPX. Negative control sections were processed by omitting primary antibody. Normal skin tissue was used as positive control for TGM-3.

### Evaluation of staining

The level of expression was assessed by semiquantitative scoring which included the overall percentage area of the lesion stained positive (0–100%), and the staining intensity (0–3). In all the cases, the expression in epithelium, endothelial cells, tumour cells and stroma were analyzed. Grading for percentage area positivity was done as follows: <10% = 0, 10–25% = 1, 25–50% = 2, 50–75% = 3, >75 = 4. For evaluating intensity, grading was done as; 0 = none, 1 = mild, 2 = moderate, 3 = strong staining. The percentage area score (0–4) was multiplied by the intensity score (0–3) and a final score was assigned, 0–4 as negative staining, 5–12 as positive staining [21]. Five best fields were taken for interpreting results of percentage area.

### Quantitative real-time PCR (qPCR) for TGM-3 gene

#### Total RNA extraction

RNA was extracted from frozen tissue samples with Trizol reagent (Invitrogen, Carlsbad, CA). RNA purification was done by DNase1 treatment (Thermo Scientific, Amplification grade). In brief, 1μg of total RNA sample was treated with 10X DNase I reaction buffer and DNase I (1U/10μl) and incubated for 30 min at 37°C followed by inactivation of DNase I with 50 mM EDTA at 65°C for 10 min. RNA was quantified by Qubit 2.0 fluorometer (Molecular Probes, Invitrogen, USA).

#### cDNA synthesis

250ng of the total RNA was subjected to reverse transcription using random hexamer primers with Revert Aid First Strand cDNA synthesis kit (Thermo Scientific), as per manufacturer’s instructions. Briefly, the 20μl reaction was performed in 3 steps. Step 1 at 25°C for 5 min, step 2 at 42°C for 1 hrs and finally step 3 at 70°C for 5min. cDNA was stored at -20°C for real time PCR.

#### Quanitative real time PCR (qPCR)

qPCR was performed using StepOne Real-time PCR system (Applied Biosystems, USA) in the presence of SYBR Green fluorescent dye according to the manufacturer’s instructions. Briefly, 20μl of the reaction mixture consisting of reverse transcribed cDNA, 2X SYBR Green master mix containing dNTPs, ROX dye and 10μM of forward and reverse primers was dispensed into a fast optical 48-well real time PCR reaction plate (Applied Biosystems, USA). The PCR primers for TGM-3 [20] was selected from a published article and synthesized by eurofins Genomics India Pvt Ltd. Primer sequences were rechecked using Primer Express software 3.0 (Applied Biosystems, USA) and checked for homology by Blast sequence analysis (National Centre for Biotechnology Information). Following primers sequences were used: β-actin (endogenous control): forward 5’-GAGACCTTCAACACCCCAGCC-3’; reverse 5’-AGACGCAGGATGGCATGGG-3’, TGM-3 forward 5’-TCAACTGGCAGACGGCCTTCA-3; reverse 5’- GTACCGTCCTATGGGTGCGCT-3’. Thermal cycle conditions consisted of on initial denaturation incubation at 95°C for 10min followed by 40 cycle of 15sec incubation at 95°C and 60sec incubation at 60°C followed by the thermal dissociation (melt curve) protocol for fluorescence detection. Gene expression level was determined using the 2^-ΔΔCt^ method using beta-actin as an endogenous control. A negative control without a template was run in parallel to assess the overall specificity of the reaction. All reactions were run in replicates. Data were presented as “relative gene expression”.

### ELISA for TGM-3

ELISA were performed on 72 serum samples of OSCC of pre chemo-radiotherapy samples and 38 post chemo-radiotherapy samples and 20 healthy controls by using TGM-3 ELISA kit (Chongging Biospes Co., Ltd) as per manufacturer’s instruction.

### Statistical analysis

Statistical analysis was performed using version 17.0 SPSS software for windows (SPSS, INC, Chicago, IL). For assessing proportional data, chi-square test was carried out. Survival curves were plotted by the Kaplan-Meier method and compared using the log-rank test. Univariate analysis of overall survival was performed by Kaplan-Meier method. Multivariate analyses of overall survival were measured by Cox proportional hazards model in a stepwise manner. For all the tests, a *P* < 0.05 was considered as statistically significant.

## Results

### Patient characteristics

The study population comprised of 96 OSCC and 32 healthy controls. The age of all patients ranged from 20 to 84 years with mean ±SD was 43.38±15.61 years (median age 50 years). Tongue was the most prominent site in OSCC (43%). Histologically well differentiated (WD) cases were 50%, moderately differentiated (MD) cases were 29.17% and poorly differentiated (PD) cases were 20.83%. Lymph node metastasis was found in 69.79% cases. 46.87% cases were of stages I-II and 53.13% cases were of stages III-IV.

### Immunohistochemical (IHC) expression of TGM-3 in OSCC and controls

**Fig 1:**
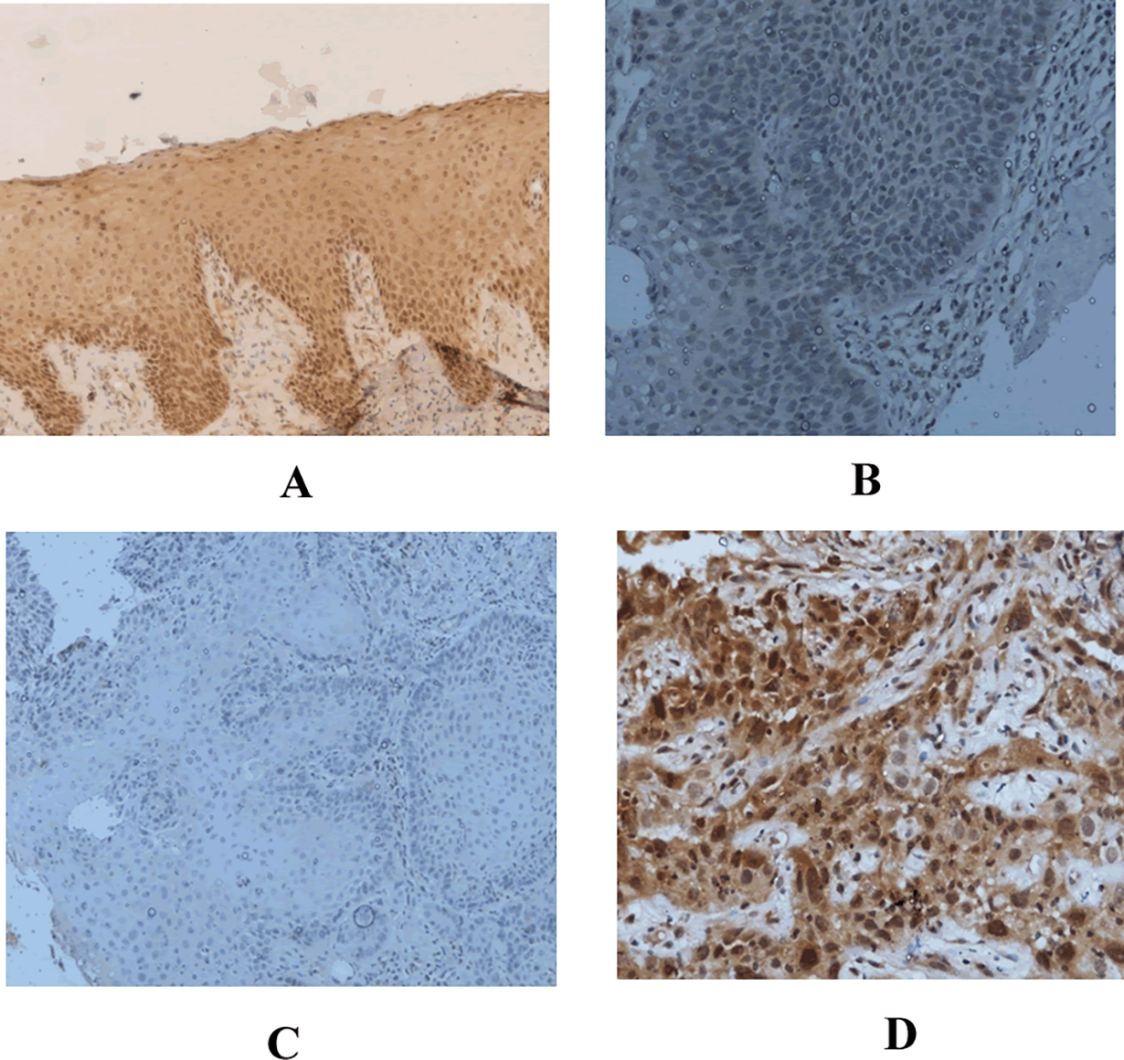
**(A-D). Immunohistochemical staining of TGM-3in case and control.** A: Strong Immunohistochemical expression of TGM-3 in healthy control (IHC X10). B: Mild immunohistochemical staining of TGM-3 in case of carcinoma in situ (IHCX 20). C: Negative immunohistochemical expression of TGM3 in tumour cells of OSCC (IHC X10). D: Strong immunohitochemical expression in OSCC well differentiated tumour (IHCX20).

Expression of TGM-3wascytoplasmic and nuclear staining expressed in keratinized layer, stratum granulosum and stratum spinosum in controls “Fig 1A” and also cytoplasmic and nuclear staining in tumour cells as shown in “Fig 1B, 1C and 1D.” In OSCC cases 76.70% cases were positive and in controls 90.62% cases were positive for TGM-3 IHC and the difference was statistically significant (p< 0.011) as shown in “Table 1”.

**Table 1:** Immunohistochemical TGM-3 expression and its association with clinicopathological characteristics.

Variables	TGM-3 Positive	TGM-3 Negative	^a^χ^2^	P-Value
**Controls**	29	3	6.46	**0.011**
**Cases**	65	31		
**Age**				
>50	29	15	0.120	0.729
≥50	36	16		
**Sex**				
Male	54	24	0.441	0.507
Female	11	7		
**Lymph node metastasis**				
Present	48	19	1.570	0.210
Absent	17	12		
**Tumour Stage**				
Stage I-II	28	17	1.16	0.280
Stage III-IV	37	14		
**Tumour Differentiation**				
WD	34	14	0.889	0.641
MD	17	11		
PD	14	6		
**Tobacco chewing habit**				
Present	45	24	0.696	0.404
Absent	20	7		
**Alcohol**				
Present	28	15	0.239	0.625
Absent	37	16		
**Smoking**				
Present	38	19	0.070	0.792
Absent	27	12		
**Tobacco+ Alcohol**				
Present	25	9	0.816	0.366
Absent	40	22		
**Tobacco+ Smoke**				
Present	23	14	0.847	0.357
Absent	42	17		
**Alcohol+ Smoke**				
Present	21	12	0.804	0.669
Absent	43	19		
**Tobacco+ Alcohol+ Smoke**				
Present	18	9	0.019	0.891
Absent	47	22		
**Response to chemo-radiotherapy treatment**				
No	6	8	9.89	**0.007**
Partial	16	1		
Complete	8	3		

^a^χ^2^ = Chi square.

### Association of Immunohistochemical expression of TGM-3 with clinico-pathological parameters

Association of TGM-3 IHC expression with clinico-pathological parameters were analyzed by using chi-square test as shown in “Table 1.” TGM-3 expression was significantly associated with response to chemo-radiotherapy treatment (p<0.007). There was no significant association of TGM-3 with age, sex, adverse oral habit, lymph node metastasis, tumour stage and differentiation.

### ELISA for serum TGM-3 in OSCC and controls

**Fig 2:**
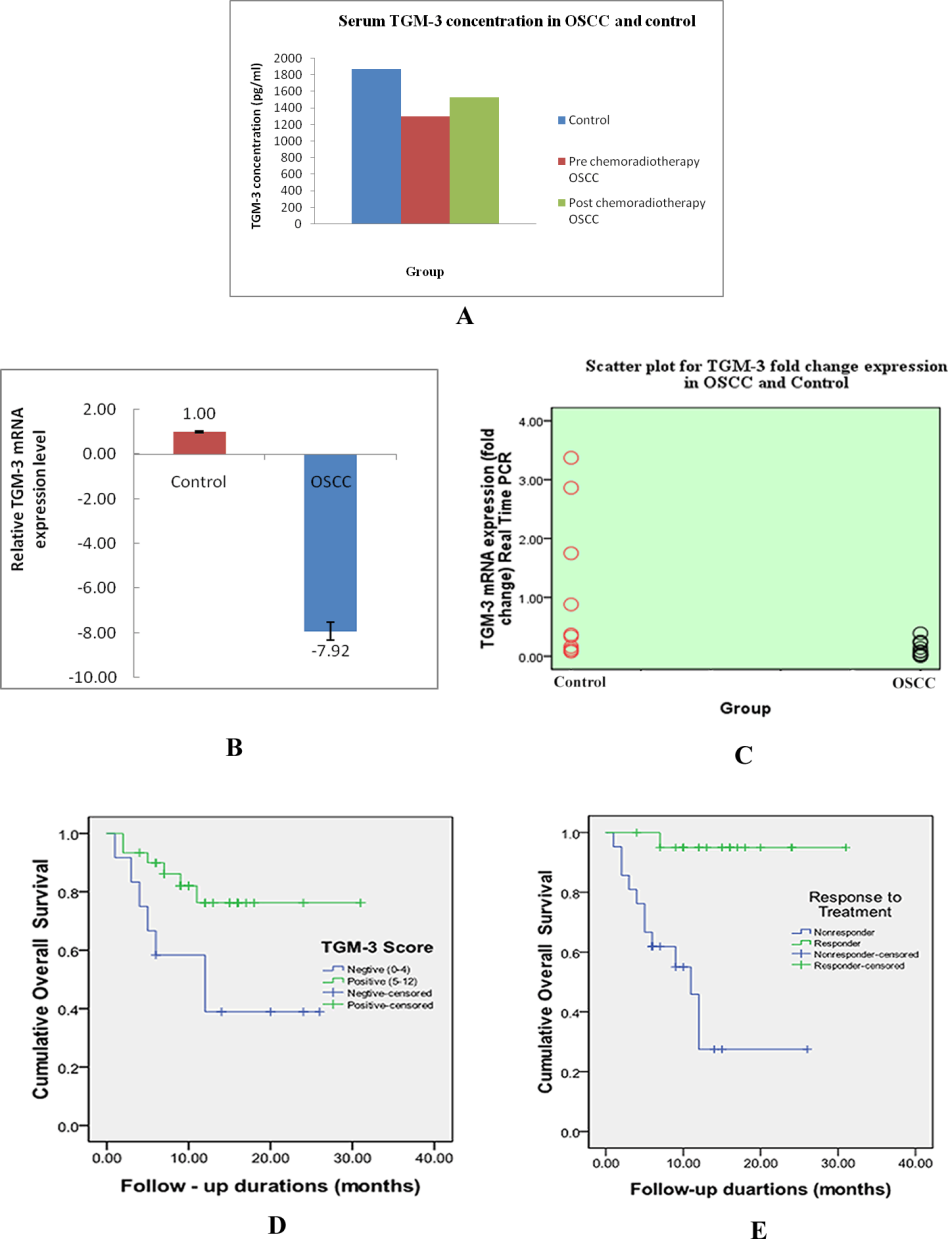
**(A-E). TGM-3 ELISA and Real Time PCR results and Overall survival curves.** A: Bar diagram for serum TGM-3 concentration in OSCC and controls. B: Bar diagram showing fold change expression of TGM-3 gene by Real Time PCR in OSCC with respect to control. C: Scatter plot for fold change expression of TGM-3 gene in OSCC and control. D: Kaplan- Meier overall survival curve in correlation with TGM-3 expression. E: Kaplan- Meier overall survival curve in correlation with response to chemo-radiotherapy treatment.

Mean serum TGM-3 levels in pre and post chemo radiotherapy samples and controls were shown in “Table 2.” Mean TGM-3 level was higher in Controls (1869.16±1377.36) than in post chemo radiotherapy OSCC (1530.64±669.33) samples and lowest in pre chemo-radiotherapy samples (1304.83±573.55) as shown in “Fig 2A.” Mean TGM-3 serum level is statistically significant in pre chemo-radiotherapy samples as compared to control (p<0.018).

**Table 2:** ELISA for TGM-3 in pre and post chemo-radiotherapy samples.

Mean serum concentration in controls (pg/ ml)	Samples	Mean serum concentration in cases (pg/ml)	Levene’s test for equality of variance between two variables	Df	P- value
1869.16±1377.36	**Pre- chemoradiotherapy Samples**	1304.83±573.55	OSCC/Controls (N = 72/20)	90	0.018
	**Post chemoradiotherapy samples**	1530.64±669.33	OSCC/Controls (N = 38/20)	56	0.162

### Quantitative real—time PCR for TGM-3 expression

Quantitative real—time PCR was done to validate the results of IHC and serum ELISA of TGM-3. It was observed that relative gene expression of TGM-3 was lower (-7.92 fold) than controls and the difference was statistically significant (p<0.04) as shown in “Table 3” and “Fig 2B and 2C”.

**Table 3:** Quantative real time expression of TGM-3 gene expression as fold of internal control gene (β- actin) in cases and controls.

Group	^a^2^-ΔΔCT^ (fold change)	SE	P-value
**Control (N = 10)**	1	0.65	
**OSCC (N = 10)**	-7.92	0.68	0.04

^a^2^-ΔΔCT^ = fold change.

### Association of TGM-3 expression with response to chemo-radiotherapy treatment

Out of 96 patients of OSCC, follow up was possible only for 42 patients who underwent chemo-radiotherapy treatment. Fifteen patients who underwent surgery, and Thirty nine were dropouts during the course of follow up, were excluded from the analysis, Out of remaining 42 patients, 14 were NR, 15 were PR and 11 patients were CR. Hence, 26 patients were included as responders and 14 as non-responders. Response to chemo-radiotherapy treatment was positively associated with overall survival (p<0.000) and TGM-3 (p<0.006) expression. Patient’s characteristic of responded and non responded cases to chemo-radiotherapy was shown in “Table 4.” Patients having positive TGM-3 expression in OSCC have better response to chemo-radiotherapy treatment.

**Table 4:** Characteristics of patients with response to chemo-radiotherapy treatment.

Variables	Responder	Non Responder	P-value
**Age**			
Mean ± SD	49.80 ± 10.94	57.64 ±12.23	
Range	29–65	40–84	
**Length of Follow- up (Months)**			
Mean ± SD	14.57± 6.46	8.14 ± 5.71	
Range	4–31	1–26	
**Sex**			
Male	18	17	0.679
Female	3	4	
**Lymph node metastasis**			
Present	15	16	0.726
Absent	6	5	
**Tumour Stage**			
Stage I-II	12	11	0.757
Stage III-IV	9	10	
**Tumour Differentiation**			
WD	2	4	0.648
MD	8	8	
PD	11	9	
**Tobacco chewing habit**			
Present	17	15	0.739
Absent	7	6	
**Alcohol**			
Present	9	10	0.757
Absent	12	11	
**Smoking**			
Present	15	13	0.513
Absent	6	8	
**Tobacco+ Alcohol**			
Present	8	7	0.747
Absent	13	17	
**Tobacco+ Smoke**			
Present	10	7	0.346
Absent	11	14	
**Alcohol+ Smoke**			
Present	8	7	0.747
Absent	13	14	
**Tobacco+ Alcohol+ Smoke**			
Present	6	6	1.000
Absent	15	15	
**Overall survival**			
Yes	20	9	**0.000**
No	1	12	
**TGM-3 expression**			
Positive	19	11	**0.006**
Negative	2	10	

### Overall survival analysis in OSCC patients

Out of 42 patients, 29 patients survived and 13 died. Overall survival was significantly associated with expression of TGM-3 (p<0.024) and response to chemo-radiotherapy treatment (p<0.000) as shown in “Fig 2D and 2E.” Other factors viz age, sex, tumour stage, differentiation, lymph node metastasis and adverse oral habit did not have statistical significant correlation with overall survival. The results of univariate and multivariate analysis with cox proportional–hazards model were shown in “Table 5.” Patients who responded to chemo-radiotherapy and were higher level of TGM-3 have better survival. The multivariate analysis showed that response to chemo-radiotherapy and TGM-3 expression were independent prognostic effect on overall survival.

**Table 5:** Univariate (log rank test) and multivariate analysis (cox proportional hazard model) of overall survival in OSCC.

Variables	Univariate analysis		Multivariate analysis		
	Log rank	P-value	Hazard ratio	^a^95%CI	P-value
**Age**	0.047	0.829			
**Sex**	0.308	0.579			
**Lymph node metastasis**	0.608	0.436			
**Tumour Stage**	0.298	0.585			
**Tumour Differentiation**	2.401	0.301			
**Tobacco chewing habit**	0.006	0.936			
**Alcohol**	1.193	0.275			
**Smoking**	1.205	0.272			
**Tobacco+ Alcohol**	0.819	0.366			
**Tobacco+ Smoke**	0.790	0.374			
**Alcohol+ Smoke**	1.105	0.293			
**Tobacco+ Alcohol+ Smoke**	1.278	0.258			
**Response to chemo-radiotherapy**	15.60	**0.000**	19.495	2.508–151.546	**0.005**
**TGM-3 IHC expression (score 0–4 vs. 5–12)**	5.066	**0.024**	3.247	1.087–9.703	**0.035**

^a^95%CI = 95% confidence interval.

## Discussion

There are several factors that influence response to radiotherapy in head and neck cancer patients, includes tumour characteristics like location, volume and tumour stage, patient characteristics like smoking status and biological factors like hypoxia and expression of DNA repair genes [22–25]. High-throughput microarray technology might be an efficient way to uncover clues to these processes and find biomarkers for the diagnosis, therapy and prognosis of HNC [26–28].

In the present study we evaluated the expression of TGM-3 both at genotypic and phenotypic level and also its serum concentration and correlated with clinico-pathological factors, response to chemo-radiotherapy treatment and overall survival for evaluating its prognostic value.

TGM-3 was down regulated in laryngeal carcinoma, esophageal carcinoma and OSCC [17–19]. In our study also TGM-3 was down regulated in OSCC as compared to controls both in tissue and sera. This finding was also confirmed by real- time PCR analysis in which down regulation (-7.92 fold change) of TGM-3 in OSCC as compared to controls. Wu et al. [20] also reported down—regulation in HNSCC as compared with adjacent normal tissues by doing oligonucleotide microarray analysis. Although TGM-3 down-regulation has been found in many cases, the molecular mechanism that causes the silencing of TGM-3 expression is not known. He et al. reported that loss of heterozygosity within and near the TGM-3 gene may cause down regulation of TGM-3 in laryngeal carcinoma [17]. Aside from genetic changes, other epigenetic alterations including DNA methylation, histone modification and non-coding RNA regulation could also cause down-regulation of TGM-3 [20].

In this study we correlated TGM-3 with clinico-pathologcal factor and found there is no correlation with age, sex, lymph node metastasis, stage and tumour differentiation. Mendez et al. [29] reported TGM-3 expression was inversely correlated with lymph node metastasis of OSCC and Wu et al. [20] found no correlation and this was most likely due to different type and number of tumour samples in different studies.

In our study we found positive correlation of TGM-3 with response to chemo-radiotherapy treatment and overall survival by univariate (p<0.000; p<0.024) and multivariate analysis (p<0.005; p<0.035). The 2- year overall survival rate for TGM-3 positive cases were 82.5% and for TGM-3 negative cases were 17.5%. The patients having positive TGM-3 expression have better responder to chemo-radiotherapy and also have better overall survival. Wu et al. [20] observed that patients whose tumours expressed a low level of TGM-3 had worse overall survival (P<0.0002) and that TGM-3 expression, by univariate and multivariate analyses, was an independent prognostic factor in patients with HNSCC. Uemura et al. [18] also reported that the 5-year disease-specific survival rate was 64.5% and 32.1%, respectively, for patients with TGM-3 positive and TGM-3 negative.

Prognosis of OSCC is still relatively poor despite the use of modern surgical techniques in combination with radio- and chemotherapy and 5 year survival rate is still between 30% and 40% in most studies [30]. Although the use of adjuvant and neoadjuvant chemotherapies has improved the survival of patients but these treatment modalities are ineffective in most patients and are associated with severe side effects. Those patients who can be completely cured by surgery alone receive unnecessary and harmful combination therapy. The response to surgery or chemo-radiotherapy treatment is variable, even when the patients are at the same clinical stage, and it should not predicted by the existing diagnostic modalities. Accurate risk stratification is therefore important to avoid potential morbidity due to over-treatment or prevent further progression of disease [18]. Currently, no effective targeted drugs are available for this type of cancer so there is a continued need for biomarker for responsiveness to treatment. Our study demonstrated that TGM-3 might be used as prognostic biomarker for OSCC patients.

Li et al. [31] demonstrated that TGM-3 can be a candidate tumour suppressor that is able to induce EC cell proliferation and migration by down regulating the NF-κB signalling pathway, indicating that TGM-3 may serve as a useful biomarker and therapeutic target for esophageal cancer treatment.

The conclusion of this study is that TGM-3 might be candidate biomarker for responsiveness to radiotherapy in OSCC patients. As this study is done on small samples size because many patients withdrawn from follow or left treatment in middle stage as these patients are economically very poor and illiterate. Hence this should be validated in larger sample size with longer follow–up of chemo-radiotherapy response.
