# Correction: Tissue and serum expression of TGM-3 may be prognostic marker in patients of oral squamous cell carcinoma undergoing chemo-radiotherapy

**DOI:** 10.1371/journal.pone.0202432

**Published:** 2018-08-09

**Authors:** Seema Nayak, M. L. B. Bhatt, Madhu Mati Goel, Seema Gupta, Abbas Ali Mahdi, Anupam Mishra, Divya Mehrotra

The third author’s contributions are incorrect. Madhu Mati Goel’s correct contributions are: Conceptualization, Data Curation, Methodology, Project administration, Resources, Supervision, Validation, Visualization, Writing–review & editing.
